# The Beginning of Metallurgy in the Southern Levant: A Late 6^th^ Millennium CalBC Copper Awl from Tel Tsaf, Israel

**DOI:** 10.1371/journal.pone.0092591

**Published:** 2014-03-26

**Authors:** Yosef Garfinkel, Florian Klimscha, Sariel Shalev, Danny Rosenberg

**Affiliations:** 1 Institute of Archaeology, The Hebrew University of Jerusalem, Mt. Scopus, Jerusalem, Israel; 2 German Archaeological Institute, Eurasia Department, Berlin, Germany; 3 Department of Archaeology, Department for Maritime Civilizations, University of Haifa, Haifa, Israel; 4 Laboratory for Groundstone Tools Research, Zinman Institute of Archaeology, University of Haifa, Haifa, Israel; University at Buffalo, United States of America

## Abstract

The beginning of metallurgy in the ancient Near East attracts much attention. The southern Levant, with the rich assemblage of copper artifacts from the Nahal Mishmar cave and the unique gold rings of the Nahal Qanah cave, is regarded as a main center of early metallurgy during the second half of the 5^th^ millennium CalBC. However, a recently discovered copper awl from a Middle Chalcolithic burial at Tel Tsaf, Jordan Valley, Israel, suggests that cast metal technology was introduced to the region as early as the late 6^th^ millennium CalBC. This paper examines the chemical composition of this item and reviews its context. The results indicate that it was exported from a distant source, probably in the Caucasus, and that the location where it was found is indicative of the social status of the buried individual. This rare finding indicates that metallurgy was first defused to the southern Levant through exchange networks and only centuries later involved local production. This copper awl, the earliest metal artifact found in the southern Levant, indicates that the elaborate Late Chalcolithic metallurgy developed from a more ancient tradition.

## Introduction

**Figure 1:**
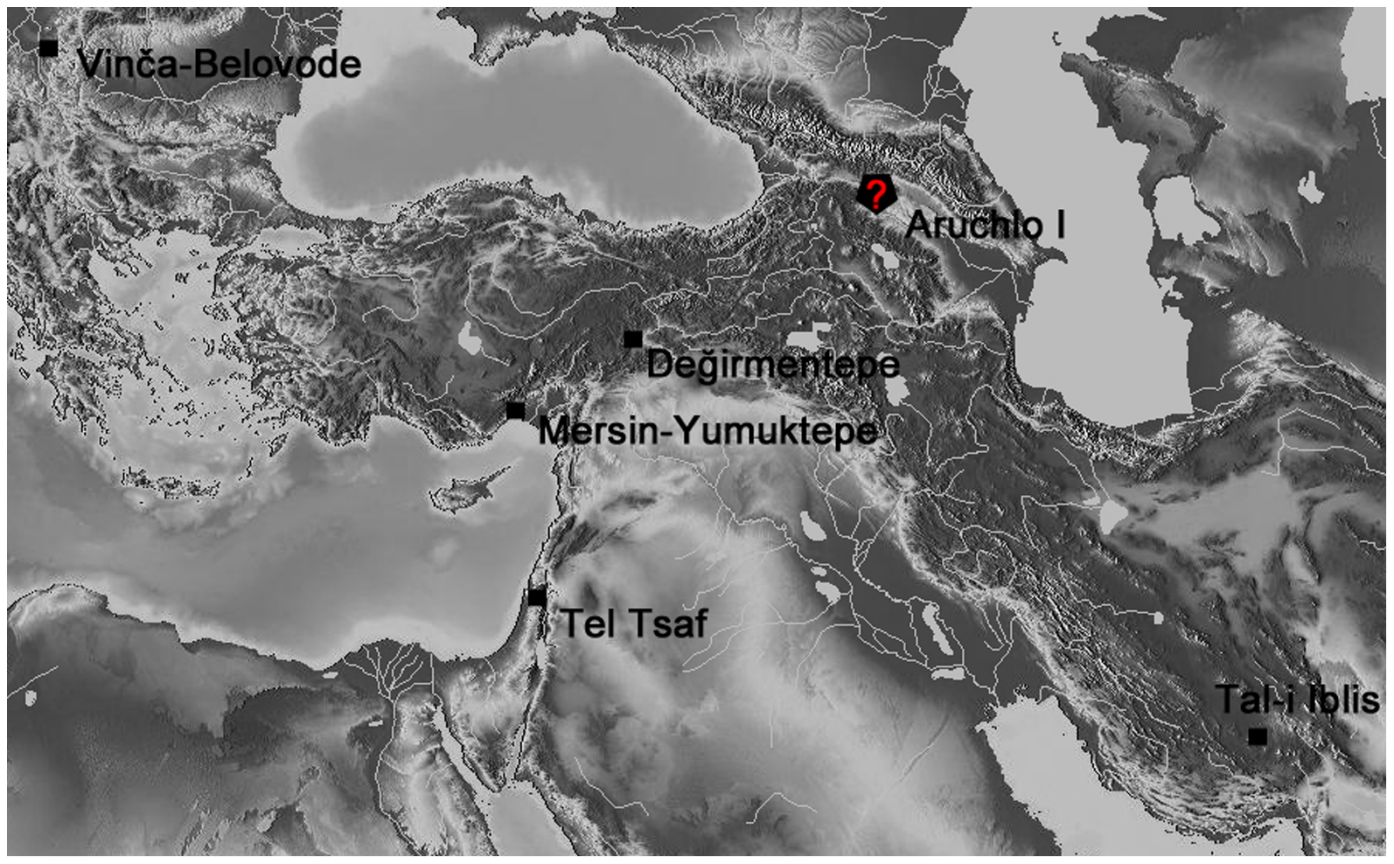
Map showing the main sites mentioned in the text: the distribution of smelted copper objects during the late 6^th^–early 5^th^ millennia CalBC.

According to the current state of research the initial appearance of copper in the southern Levant is dated to the Late Chalcolithic period (Ghassulian culture, ca. 4500–3800 CalBC, all dates are calibrated). The elaborate assemblage of prestige items from the Cave of the Treasure in Nahal Mishmar, displaying lost wax casting technology, and the electrum and gold items from Nahal Qanah cave place the southern Levant as a major center of metallurgy in the Near East [1]–[3]. However, no data has been available until now about the background of this technology in the region. A recently discovered cast metal awl, found in a grave associated with a primary burial inside a built mud-brick silo at Tel Tsaf in the central Jordan Valley, Israel (Figure 1), reveals new evidence for these significant early stages.

Tel Tsaf was surveyed and tested in the past [4]–[5] and four large-scale excavation seasons were conducted between 2004 and 2007 [6]–[7]. Nearly 800 m^2^ were opened, and a poorly preserved layer of Late Byzantine–Early Islamic occupation was noted just below the topsoil. The main occupation of the site is composed of a densely built Middle Chalcolithic settlement, with a few stratigraphic phases, radiometrically dated between 5100–4600 CalBC. For the archaeology of the southern Levant, the term “Middle Chalcolithic” refers to the time-span between the Wadi Rabah Culture (Early Chalcolithic) and the Ghassulian Culture (Late Chalcolithic), and has been explained in detail elsewhere [8]–[9].

The complex mud-brick architectural settings include courtyard buildings combining rectilinear rooms, rounded rooms and silos, as well as many cooking facilities. Four burials were uncovered. Two of these were found inside two distinct silos, while the other two graves were found in proximity to two other silos. The silos uncovered in courtyard structures reached a storage capacity estimated at 15–30 tons of grain, far beyond the yearly need of a nuclear family, a clear indication of the accumulation of surpluses on a scale unprecedented in the ancient Near East. The material culture of the site presents rich assemblages of elaborately painted pottery, over 2,500 beads made of ostrich egg-shell which were found mainly in two distinct concentrations, about 100 stone beads, many obsidian items originating in Anatolia or Armenia, four Ubaid pottery shards imported from either north Syria or Mesopotamia and a Nilotic shell from Egypt. No other known site of this period exhibits long-distance connections in a similar dimension. An exceptionally rich faunal assemblage was also noted, dominated by large numbers of cattle and pigs [10].

We present here the copper awl found in one of the silo-graves at Tel Tsaf and discuss the characteristics of this unique artifact, its chemical composition and its significance for early metallurgy in the Near East. Tel Tsaf exhibits an exceptionally rich community, with storage capacity previously unknown in the prehistoric Near East, accumulation of wealth, and long distance exchange networks with different parts of the ancient Near East. The available dating from Tel Tsaf and the item context suggest that this awl is the earliest known copper artifact in the southern Levant, suggesting that cast metal technology was introduced to the southern Levant centuries before the onset of the full-blown Ghassulian Chalcolithic.

## Results

**Figure 2:**
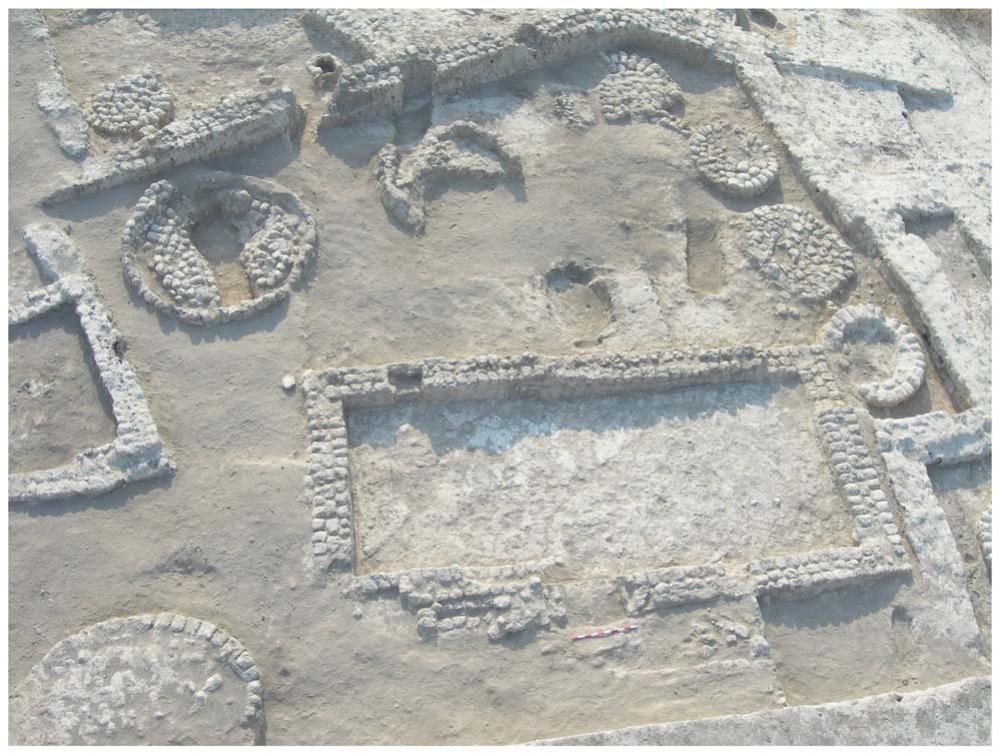
General view of Courtyard Building 1. The silo in which the burial was found is located in the upper left corner.

**Figure 3:**
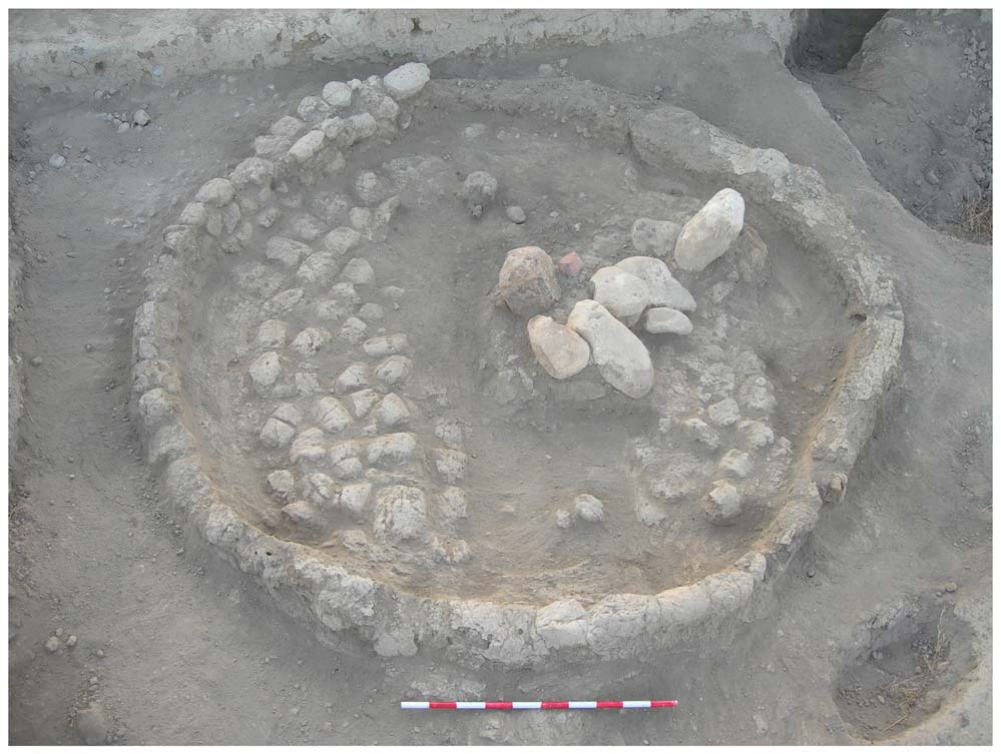
General view of the rounded silo, with large stones at its center. At this stage the burial had not yet been uncovered.

**Figure 4:**
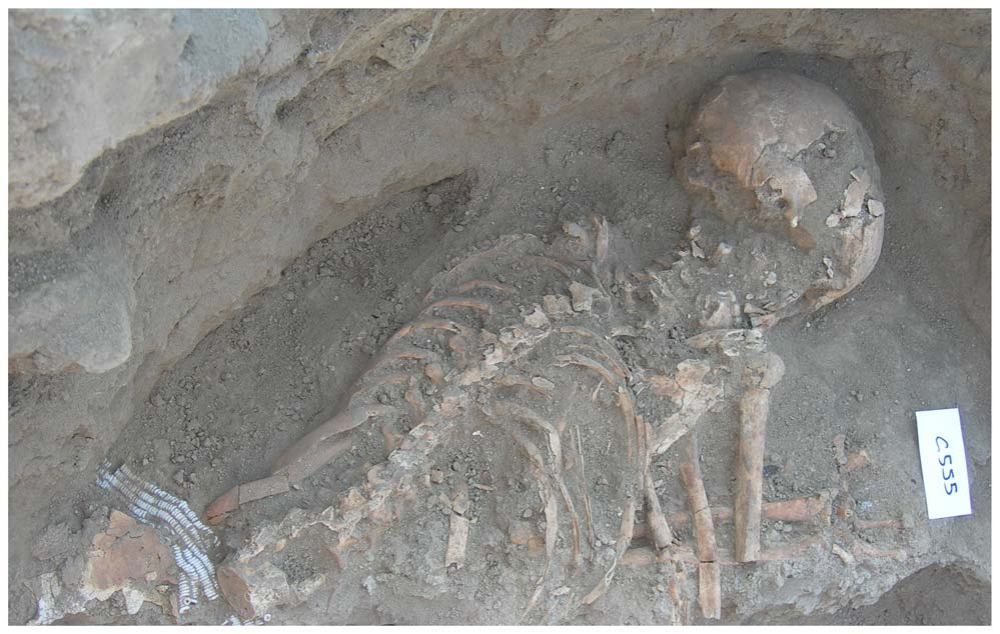
Close-up of the burial, with the belt of 1,668 beads on its pelvis.

The metal awl was discovered in the fills of Grave C555, set within Silo C339 in the northeast corner of the courtyard of Building I, during the 2007 excavations (Israel Antiquities Authority License number: 2007/G38) (Figures 2–3). The burial includes an articulated skeleton of an adult female who was approximately 40 years old [7]. The grave was oriented north–south (with the skull in the south). The head was turned to the right, the knees were flexed and the arms were slightly flexed at the elbow. The burial was found with a necklace encompassing 1,668 ostrich egg-shells beads placed on the frontal part of the pelvis and arranged in six rows (Figure 4). The grave was cut into the mud-brick floor of the silo and after the interment it was filled and covered with large cobbles.

The process of excavating the silo and identifying its components involved several stages. First the rounded peripheral wall was noted. Later, large stones were recognized in the center of the silo. Further excavations uncovered the silo floor, which was paved with bricks. At the center of the silo, below the stones, the pavement was missing. Further excavations in the area where the paving was missing uncovered a human burial. The copper awl was uncovered while sieving the sediment collected during the exposure of the burial (i.e. the sediments that covered the burial). It was probably hidden by sediment that enveloped it and that sediment was removed during the sieving process. As the awl was found in the sediment collected in association with the skeleton, it was apparently a burial offering.

The location of the awl was nearly 1.5 m. below the original topsoil. It was found in association with a rich burial, in the middle of a built silo, and the burial pit was covered with stones. No other occupational phases were found above or below the burial. Thus, the awl was found in a secure context. As the burial was rich in body ornaments (a belt of 1668 beads) the awl was probably directly associated with the burial.

**Figure 5:**
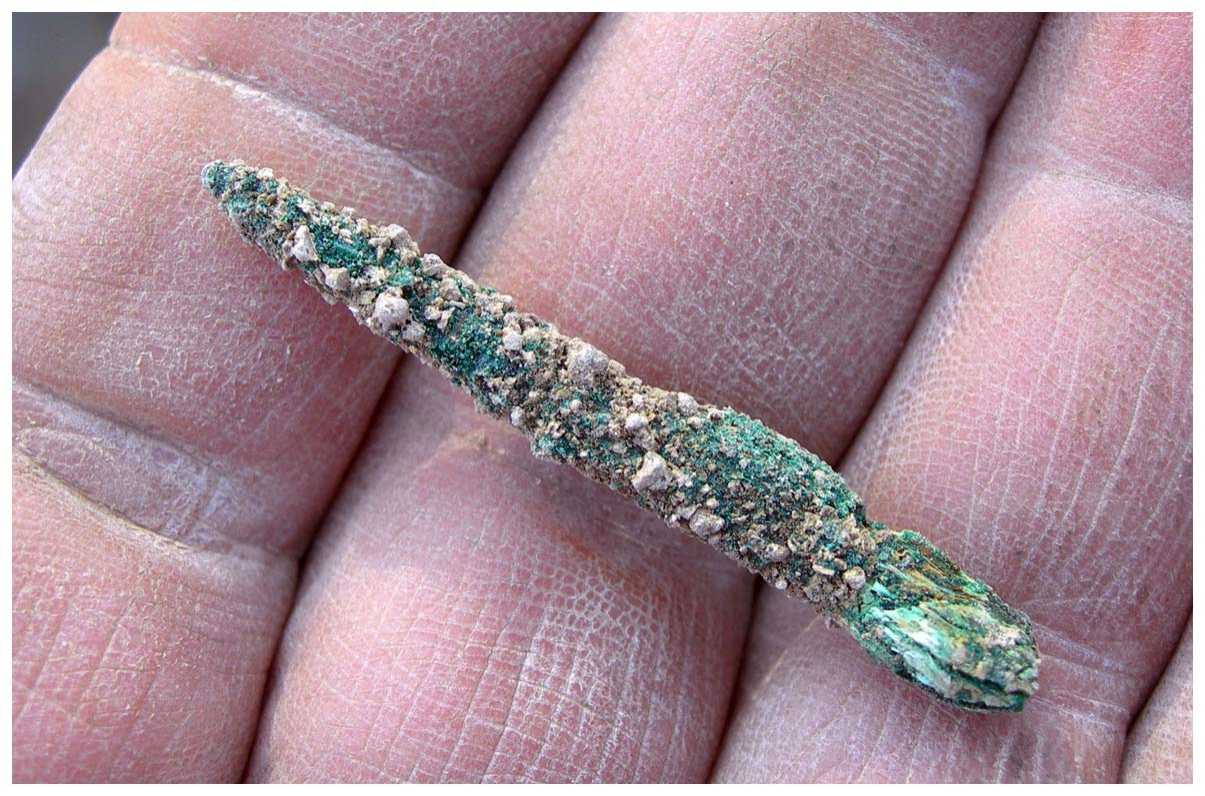
The metal awl from Tel Tsaf upon discovery.

The metal object is an elongated pin made of cast copper, with a rounded cross-section (Figure 5). It is 41 mm. long and its maximum diameter (near the base and at the middle of its length) is 5 mm. The diameter of the awl near its tip is 1 mm. The color of the outside is green due to oxidization and corrosion, while the core is reddish. The narrower tip bears signs of rotational movement and remains of a wooden handle were noted on the base, on the opposite end.

The awl is totally corroded with no original metal left. The chemical composition of the corroded metal was analyzed on the cleaned, relatively flat, corroded sections of the pin on both broken inner surfaces. Due to the corroded state of this object we could not observe the original microstructure; no rare remains of the original production process visible, nor the original relative quantities of the various elements preserved. The corroded inner sections of both fractures were analyzed by a portable Niton ED-XRF using a beam size of 3 mm. in diameter. The results (Table 1) show copper corrosion with 6% tin (Sn) and 0.8% arsenic (As) and low traces of lead (Pb) and Iron (Fe).

**Table 1:** Results of portable Niton ED-XRF analysis (beam size: 3 mm in diameter).

No.	1	2	3	4	5	6
**Reading No**	516	519	520	522	523	524
**Sample Location**	blunt edge section	blunt edge section	blunt edge polished section	blunt edge polished section	sharp edge polished section	Cu control
**Duration**	123,13	121,93	122,95	121,94	121,62	121,78
**Units**	%	%	%	%	%	%
**Cu**	92,431	92,497	95,197	93,052	89,027	97,227
**Cu Error**	0,046	0,046	0,029	0,044	0,132	0,087
**Sn**	6,247	6,227	3,584	5,738	7,079	< LOD
**Sn Error**	0,033	0,033	0,018	0,031	0,094	0,027
**Sb**	< LOD	< LOD	< LOD	< LOD	< LOD	< LOD
**Sb Error**	0,024	0,025	0,019	0,025	0,065	0,038
**As**	0,841	0,836	0,569	0,813	0,906	< LOD
**As Error**	0,017	0,017	0,009	0,016	0,048	0,008
**Bi**	< LOD	< LOD	< LOD	< LOD	< LOD	< LOD
**Bi Error**	0,005	0,005	0,003	0,005	0,014	0,009
**Pb**	0,08	0,074	0,055	0,067	0,114	< LOD
**Pb Error**	0,006	0,006	0,004	0,006	0,02	0,008
**Zn**	0,153	0,107	0,109	0,068	< LOD	< LOD
**Zn Error**	0,016	0,016	0,01	0,015	0,084	0,056
**Ni**	< LOD	< LOD	< LOD	< LOD	< LOD	< LOD
**Ni Error**	0,014	0,013	0,01	0,013	0,038	0,022
**Co**	< LOD	< LOD	< LOD	< LOD	< LOD	< LOD
**Co Error**	0,008	0,007	0,005	0,007	0,022	0,011
**Fe**	0,196	0,197	0,369	0,218	0,213	0,019
**Fe Error**	0,008	0,008	0,006	0,008	0,023	0,009

The ratios between elements are not as they were in the original metal and may have been altered during the corrosion process. Still, the appearance of tin, even if highly enriched in the corrosion process, raises important questions. Until now, copper items of such chemical composition have not been found in the Late Chalcolithic or the Early Bronze Age of the southern Levant, nor does the metal composition of this object, which includes tin, fit the known compositions of local native copper. In fact, items with similar chemistry have been documented in the southern Levant up to now only from the Middle Bronze Age, the 2^nd^ millennium CalBC, and later periods. Thus, not only does the Tel Tsaf awl predate all known metals in the southern Levant by several centuries, it also predates all known tin bronze items in this region by ca. 3,000 years.

## Discussion

According to the current understanding of the archaeological record, the usage of copper minerals was developed in three main stages in the Near East. During the first stage, natural Malachite chunks were used for the production of pigments and ornaments, as early as the Epipalaeolithic of Anatolia [11]–[12]. Beads made of green minerals have been found in a few Natufian and Pre-Pottery Neolithic sites in the southern Levant as well [13]. Malachite chunks were uncovered at Hallan Çemi and Çayönü Tepesi, both in southeastern Anatolia, and dated to ca. 10500–8800 CalBC [14]–[15].

At the second stage, a major development is reported in Anatolia during the Pre-Pottery Neolithic B of the 7^th^ millennium CalBC when beads and other decorative objects were made from hammered native copper. Such items have, for example, been reported at Aşıklı Höyük [16] and Nevali Çori [17]. Some finds from Çayönü show possible evidence of early heating during the production process [18]. Comparable finds are known from the Pre-Pottery Neolithic B site of Tell Halula in Syria [19] and at Ali Kosh in Iran [20]. To date, no metal finds have been reported from Pre-Pottery Neolithic B sites in the southern Levant. Native copper hammering is usually characterized by small ornamental items. However, around 6000 CalBC examples of larger objects are known from Çan Hassan [12]. Similar techniques are skillfully used to produce elaborate objects, for instance at Tepe Yahya VII in Iran [21].

The third phase, dated to the late 6th millennium CalBC, includes smelting and melting of ores to extract copper, a much more complicated process than simply using native copper: A single error in the complex and lengthy production chain will ruin the finished product. This was discovered at various sites in the Balkans [22]–[23] and in southern Anatolia, at Mersin-Yumuktepe layer XVI and Değirmentepe [24]–[25], [12]. Such pieces can be identified by significant amounts of trace elements such as antimony, cobalt, lead, arsenic and nickel resulting from the smelted copper ore [26]. At Tal-i Iblis in Iran crucible smelting is dated to the first quarter of the 5^th^ millennium CalBC [26]–[27].

The earliest extractive metallurgy in the southern Levant is typically connected with the Late Chalcolithic period (ca. 4500–3800 CalBC) and reliable ^14^C dates show that the prestige items were manufactured as early as 4350–4250 CalBC [28]–[29]. In the southern Levant, an extremely elaborate tradition developed using lost-wax casting of combining copper ores with high antimony or nickel content with arsenic rich ores to cast prestige objects, as known at various sites in southern Israel [30]–[33]. Simultaneously, unalloyed copper was used in simple open molds for the production of objects like axes, chisels and awls. Evidence for the earliest electrum and gold was found in the burial cave of Nahal Qanah [2]. Petrography of the clay cores or the remains of moulds of items from Nahal Mishmar suggest that the casting of the prestige items took place locally, in the southern Levant [34].

The elaboration of south Levantine Late Chalcolithic metallurgy means that with our current archaeological record, the peak of the technical evolution of copper metallurgy is set at its beginning. In our opinion, this suggests that large parts of the technological evolution of metallurgy have not yet been discovered. Indeed, the Tel Tsaf awl dated to ca. 5100–4600, centuries earlier, would thus fill an important gap in the picture. However, the high percentage of tin in the awl could be used to argue that the item was an intrusive object from a much later period—although during the excavation no disturbances were documented and the context was sealed by mudbricks and stone slabs and cobbles.

Recently, new data on very early copper artifacts in the northeastern Near East and the Balkans indicate that tin was found in some of the early metal items known in this area [35]–[37]. Another example comes from the Late Neolithic mound of Aruchlo I in Georgia, 5800–5300 CalBC [38]. This is a heavily corroded, small, ring-shaped bead, and therefore it is unclear whether it was cast or hammered. XRF-analysis identifies copper, iron, arsenic and a larger amount of tin. It was suggested that the object was made from a polymetallic raw-material, i.e. a natural copper-tin alloy [38]–[39]. A final analysis of the bead recently confirmed a copper-based (84.991%) alloy with high amounts of tin (8.350%) as well as arsenic (3.016%) and iron (3.643%) [40].

Even though it could be argued that the items from Aruchlo I and Tel Tsaf are later intrusions which were brought into earlier archaeological layers by post-depositional processes, this seems to be less plausible because there are no known later settlement traces at either site. Since artificial alloying would also be very improbable at such an early time, a natural copper-tin alloy is, at the moment, an interpretation worth considering. Copper sources with a natural tin-copper alloy are known *inter alia* in Mušiston, Tajikistan [41]–[42]. The easy availability of tin and the knowledge of natural tin-copper alloys could be one reason why the alloying of tin-bronze took place in the Caucasus significantly earlier than in neighboring regions, namely in the 4^th^ millennium CalBC [43].

If one accepts the Aruchlo I bead as non-intrusive, it demonstrates the possibility of the extraction and use of natural tin-copper alloys as early as the 6^th^ millennium CalBC. This, in turn, also opens the possibility that the item from Tel Tsaf was made from a natural tin-copper source and transported to the Jordan Valley via long-distance exchange networks, which also brought obsidian, groundstone items and other goods from Armenia, Anatolia and Syria through the Levantine Corridor [44]. If this is the case, then the copper awl from Tel Tsaf could have reached the site through exchange. The context and typology of the awl support the interpretation that the artifact belongs to the Middle Chalcolithic period:

### 1. General context

The Middle Chalcolithic site of Tel Tsaf exhibits an extremely wealthy community, with large-scale silos, unprecedented in the ancient Near East. In addition, no evidence was found for human activity at Tel Tsaf between the Middle Chalcolithic period and the Late Byzantine–Early Islamic periods.

### 2. Immediate context

The copper awl was a grave good, associated with a primary undisturbed burial. The skeleton was found in articulation, adorned with a bead necklace placed on the corpse or worn by the deceased. The ostrich-egg-shell beads found on the deceased are morphologically similar to hundreds of beads found on the floor of the same courtyard, stressing that the grave (and thus the copper awl) and the courtyard, are contemporaneous. In addition, the grave was dug in the center of a built silo base, protected from all sides by the mud bricks, and was covered by stone cobbles. No rodent dens were documented during the excavation of the grave. Very richly furnished graves are not unknown from the Chalcolithic; gold and copper items were included as grave goods, for instance, in prestige burials of the Late Chalcolithic such as Nahal Qanah and Peqi’in caves [45], [2].

### 3. Awl typology

The copper awl from Tel Tsaf is typologically comparable to others from 5^th^ millennium CalBC Ubaid sites in the Near East: Tepe Gawra Layer XIII and Ugarit Layer IIIb [46]–[47]. In addition, two similar copper awls were found in Israel at Late Chalcolithic sites: Beersheba and Shiqmim [48]–[49]. The typology of the Tel Tsaf awl thus fits well into the corpus of known copper artifacts of the Chalcolithic period in the Near East. On the other hand, such awls are not a common item in later periods.

In addition, it should be noted that the Middle Chalcolithic period was recognized as a cultural phase only rather late in the history of research [8]–[9]. It is poorly researched and the few sites known from this period in the southern Levant were excavated on a very limited scale. If copper items were found in contemporary sites at Tepe Gawra and Ugarit, similar items are to be expected in Middle Chalcolithic sites in the southern Levant.

## Conclusions

The copper awl from Tel Tsaf is a hitherto exclusive example of metallurgy in the southern Levant during the late 6^th^–early 5^th^ millennia CalBC. The available contextual evidence clearly indicates that the awl, the grave and the building all belong to a single phase in the Middle Chalcolithic period. A number of implications emerge from this conclusion. First, it suggests that the inhabitants of the southern Levant were exposed to copper metallurgy as early as the late 6^th^–early 5^th^ millennia CalBC and clearly much before the emergence of the Late Chalcolithic Ghassulian culture in the southern Levant. It seems possible that the Tel Tsaf awl was the result of smelting and melting, but the poor preservation and heavy corrosion of the object make the chemical analysis difficult to interpret and therefore leave this important question yet unanswered. Second, metallurgical technology was spread by diffusion from the north [21], [24]; initially, imported artifacts arrived through exchange networks and only later was metal produced locally.

This suggests that the elaborate Late Chalcolithic metallurgy was the product of a longer tradition. Thus, the fact that only a single item has thus far been found in such an early context may indicate primarily the current state of research and that greater emphasis should be placed upon the study of the Middle Chalcolithic period. Third, the fact that the copper awl was found in the most elaborate burial of its period in the entire Levant suggests that metal items were perceived as rare prestige goods. Finally, it appears that the occupants of Courtyard Building I belonged to a family or selected group within the community that controlled local cultivation and storage of grain as well as long-distance trade. This aggregation of wealth in the form of surplus accumulation by specific segments of the community may also have led to or stemmed from trade in exotic items obtained from remote sources over 1,000 kilometers from Tel Tsaf.

## Materials and Methods

The Tel Tsaf Copper awl (Item #1-Basket number C1029) was found during the 2007 Hebrew University of Jerusalem excavations at the Tel (Israel Antiquities Authority License number: 2007/G38), directed by Prof. Y. Garfinkel [6], [7], who provide the material for the analysis. The item is presently stored at the Institute of Archaeology, Hebrew University in Jerusalem. All necessary permits were obtained for the described study, which complied with all relevant regulations.
