# Correction: The Beginning of Metallurgy in the Southern Levant: A Late 6^th^ Millennium CalBC Copper Awl from Tel Tsaf, Israel

**DOI:** 10.1371/journal.pone.0096882

**Published:** 2014-04-28

**Authors:** The PLOS ONE Staff

There is a typo in the penultimate sentence of the Abstract. The word “defused” should be replaced with the word “diffused.” Please see the correct sentence below:

“This rare finding indicates that metallurgy was first diffused to the southern Levant through exchange networks and only centuries later involved local production.”
